# Clinical effects of Lewy body pathology in cognitively impaired individuals

**DOI:** 10.1038/s41591-023-02449-7

**Published:** 2023-07-18

**Authors:** Corinne Quadalti, Sebastian Palmqvist, Sara Hall, Marcello Rossi, Angela Mammana, Shorena Janelidze, Sofia Dellavalle, Niklas Mattsson-Carlgren, Simone Baiardi, Erik Stomrud, Oskar Hansson, Piero Parchi

**Affiliations:** 1 IRCCS, Istituto delle Scienze Neurologiche di Bologna (ISNB), Bologna, Italy; 2 Clinical Memory Research Unit, Department of Clinical Sciences, Malmö, Lund University, Lund, Sweden; 3 Memory Clinic, Skåne University Hospital, Malmö, Sweden; 4 Neurology Clinic, Skåne University Hospital, Lund, Sweden; 5 Wallenberg Center for Molecular Medicine, Lund University, Lund, Sweden; 6 Department of Biomedical and Neuromotor Sciences, University of Bologna, Bologna, Italy

**Keywords:** Alzheimer's disease, Dementia, Parkinson's disease

## Abstract

There is poor knowledge about the clinical effects of Lewy body (LB) pathology in patients with cognitive impairment, especially when coexisting with Alzheimer’s disease (AD) pathology (amyloid-β and tau). Using a seed amplification assay, we analyzed cerebrospinal fluid for misfolded LB-associated α-synuclein in 883 memory clinic patients with mild cognitive impairment or dementia from the BioFINDER study. Twenty-three percent had LB pathology, of which only 21% fulfilled clinical criteria of Parkinson’s disease or dementia with Lewy bodies at baseline. Among these LB-positive patients, 48% had AD pathology. Fifty-four percent had AD pathology in the whole sample (17% of mild cognitive impairment and 24% of patients with dementia were also LB-positive). When examining independent cross-sectional effects, LB pathology but not amyloid-β or tau, was associated with hallucinations and worse attention/executive, visuospatial and motor function. LB pathology was also associated with faster longitudinal decline in all examined cognitive functions, independent of amyloid-β, tau, cognitive stage and a baseline diagnosis of dementia with Lewy bodies/Parkinson’s disease. LB status provides a better precision-medicine approach to predict clinical trajectories independent of AD biomarkers and a clinical diagnosis, which could have implications for the clinical management of cognitive impairment and the design of AD and LB drug trials.

## Abstract (web summary)

Prospective and longitudinal analyses of patients with cognitive impairment reveal that in vivo detection of Lewy body pathology is independently associated with hallucinations, worse attention/executive, visuospatial and motor function and predicted future cognitive decline.

## Main

Neurodegenerative diseases are a leading health problem, given their devastating effects on quality of life, their high prevalence and the progressive global increase in life expectancy, which will increase their prevalence even more. The development of biomarkers allowing the in vivo detection of the different neurodegenerative pathologies underlying cognitive or motor decline is a crucial step for a timely diagnosis, accurate prediction of disease progression and the stratification of patients for clinical trials^1^. Neurodegeneration is often characterized by accumulation of misfolded proteins, where the most common are amyloid-β (Aβ) and neurofibrillary tau pathology in AD, the leading cause of dementia worldwide. The second most common proteinopathy in dementias is LB pathology, which is characterized by intracellular aggregates of misfolded α-synuclein (α-syn) forming LBs and Lewy dystrophic neurites that can manifest as both dementia with LB (DLB) and Parkinson’s disease (PD), collectively referred to as Lewy body disease (LBD)^1,2^. Cognitive impairment due to neurodegenerative diseases is often multifactorial and co-pathologies may contribute to the clinical phenotype, disease progression and response to treatment. For example, AD neuropathological changes are frequently seen together with LB pathology^3^, but it is unclear how this affects clinical phenotypes, especially regarding longitudinal trajectories of different cognitive and motor functions in different clinical disease stages. Biomarkers that identify a range of different proteinopathies in vivo may provide a precision-medicine approach to the diagnosis and prognosis of patients with neurodegenerative diseases. Positron emission tomography (PET) imaging and cerebrospinal (CSF) markers detecting Aβ and tau pathology in AD have been increasingly used to provide in vivo confirmation of AD pathology^1,4^. The development of in vitro seed amplification assays (SAAs) that indirectly detect misfolded α-syn in CSF and other tissues has recently provided a pathology-specific biomarker for LBD^5^. Notably, CSF analysis of α-syn seeding activity by SAA has shown very high diagnostic accuracy in detecting LB pathology in studies validated by postmortem neuropathological data^6–9^ and for clinically diagnosed PD and DLB^7,8,10–12^. The availability of a biomarker for LB pathology offers a new opportunity for the characterization of neurodegenerative pathologies underlying cognitive impairment and can be used to evaluate their relative contribution to phenotypic expression and disease progression. Adding the LB status to the established Aβ and tau biomarker profile^13^, we aimed to study the independent effects of LB and AD pathologies on cognitive and neurological deficits in memory clinic patients with mild cognitive impairment (MCI) or dementia from two well-characterized cohorts (the Swedish BioFINDER-1 (*n* = 398) and BioFINDER-2 (*n* = 485) studies).

## Results

### Participants and prevalence of AD and LB pathologies

**Fig. 1:**
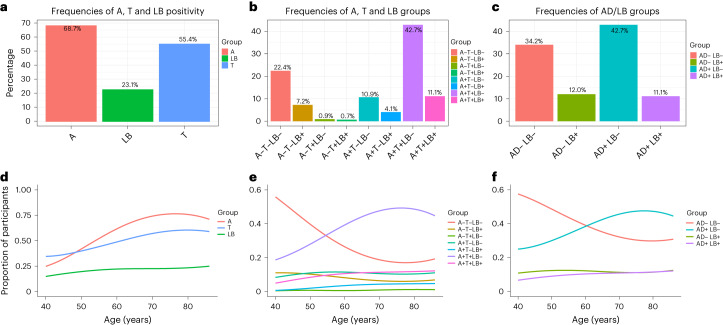
Prevalence of Aβ, tau and LB pathology. **a**, Prevalence of Aβ (A), tau (T) and LB positivity. **b**, Prevalence of A/T/LB groups (number of participants is shown in Extended Data Table 1). **c**, Prevalence of AD/LB groups. **d**–**f**, Proportions of these groups with increasing age. Percent is calculated based on the study population of 883 patients. Percentages in **a** add up to more than 100 because one can be positive in more than one biomarker. Note that AD positivity refers to being both Aβ and tau positive, whereas LB positivity refers to being α-syn SAA-positive. In **d**, using age as independent variable and pathology as dependent in logistic regression models, age had an odds ratio (OR) of 1.023 (95% confidence interval (CI) 1.001–1.047) for LB, 1.048 (95% CI 1.027–1.069) for Aβ and 1.035 (95% CI 1.016–1.055) for tau.

Aβ pathology was determined using CSF Aβ42/40, tau pathology using tau-PET or CSF phospho-tau217 (P-tau217) and LB pathology using CSF α-syn real-time quaking-induced conversion (RT-QuIC), an established SAA. AD positivity was defined as being Aβ and tau positive according to the criteria of the National Institute of Aging-Alzheimer’s Association^13^ and the International Working Group^4^ and LB positivity as being α-syn SAA-positive; AD and LB positivity refer to the biomarker status, not the clinical diagnoses, which were determined differently and not included in any statistical analyses (Methods)^14^. Biomarker status was determined at the study baseline. The prevalence of Aβ (A), tau (T) and LB, as well as different combinations of biomarker positivity, is shown in Fig. 1a–c with further patient characteristics in Table 1 and Extended Data Table 1. In patients with cognitive impairment, 302 (34%) had no AD or LB pathology (AD−/LB−). This group consisted clinically mostly of vascular cognitive impairment, frontotemporal dementia and other non-AD and non-LBD causes of cognitive impairment (Extended Data Table 1). Overall, 204 patients (23%) had LB pathology and 475 (54%) had AD pathology. LB pathology occurred with similar prevalence as isolated pathology (AD−/LB+, *n* = 106; 12%) and as co-pathology with AD (AD+/LB+, *n* = 98; 11%). In the AD+ group, 36 (17%) patients with MCI and 62 (24%) with dementia were also LB+. At baseline, 21% of LB+ participants fulfilled the clinical criteria for PD^15^ (*n* = 14) or DLB (*n* = 28) (refs. ^16, 17^) and 49% (*n* = 232) of AD+ patients fulfilled the clinical criteria for AD (MCI or dementia stage) (Table 1). The prevalence of follow-up diagnoses is shown in Extended Data Table 1 (Methods provides the diagnostic procedure). AD and LB pathologies increased with age, but this was less evident for LB pathology (Fig. 1d–f).

**Table 1:** Characteristics of the AD/LB groups

Variable	AD−/LB− (*n* = 302)	AD−/LB+ (*n* = 106)	AD+/LB− (*n* = 377)	AD+/LB+ (*n* = 98)	Total (*n* = 883)
**Age, years**	71 (7.8)	73 (6.2)	73 (7.0)	74 (6.5)	73 (7.2)
**Education, years**	11 (3.6)	12 (3.6)	11 (4.0)	12 (4.3)	12 (3.9)
**Sex, n females**	123 (40.7%)	28 (26.4%)	203 (53.8%)	50 (51.0%)	404 (45.8%)
**MCI,** ***n***	213 (70.5%)	55 (51.9%)	177 (46.9%)	36 (36.7%)	481 (54.5%)
**Dementia,** ***n***	89 (29.5%)	51 (48.1%)	200 (53.1%)	62 (63.3%)	402 (45.5%)
**MMSE, points**	26 (3.5)	25 (4.4)	24 (4.3)	23 (4.9)	24 (4.3)
**Global cognition (*z*** **score)**	−2.0 (1.1)	−2.2 (1.0)	−2.6 (1.1)	−3.0 (1.3)	−2.3 (1.2)
**Memory (*z*** **score)**	−2.4 (1.4)	−2.5 (1.2)	−3.2 (1.1)	−3.3 (1.1)	−2.8 (1.3)
**Attention/executive (*z*** **score)**	−1.4 (1.3)	−2.1 (1.8)	−2.0 (1.8)	−2.3 (1.8)	−1.8 (1.7)
**Motor function (*z*** **score)**	−1.2 (1.5)	−1.8 (1.6)	−0.78 (1.3)	−1.0 (1.3)	−1.1 (1.4)
**Hallucinations,** ***n***	15 (6.8%)	20 (26.7%)	30 (12.0%)	6 (8.8%)	71 (11.0%)
**Signs of REM sleep disorder,** ***n***^**a,b**^	25 (8.3%)^b^	27 (25.5%)^b^	11 (2.9%)^b^	7 (7.1%)^b^	70 (7.9%)^b^
**Clinical baseline diagnosis**					
AD	18 (6.0%)	8 (7.5%)	187 (49.6%)	45 (45.9%)	258 (29.2%)
DLB	1 (0.3%)	21 (19.8%)***	2 (0.5%)	7 (7.1%)***	31 (3.5%)
PD	1 (0.3%)	12 (11.3%)***	0 (0%)	2 (2.0%)***	15 (1.7%)
VaD	17 (5.6%)	3 (2.8%)	5 (1.3%)	3 (3.1%)	28 (3.2%)
FTD or tauopathy^c^	26 (8.6%)	5 (4.7%)	0 (0%)	0 (0%)	31 (3.5%)
Other or not yet determined etiology^d^	239 (79.1%)	57 (53.8%)	183 (48.5%)	41 (41.8%)	520 (58.9%)
**CSF Aβ42/Aβ40**	0.087 (0.027)	0.088 (0.026)	0.042 (0.011)	0.043 (0.012)	0.063 (0.030)
**CSF P-tau217 (pg** **ml**^**−1**^**)**	6.6 (6.5)	6.4 (2.9)	42 (26)	36 (27)	28 (27)
**Tau-PET (SUVR)**^**b,e**^	1.2 (0.11)	1.2 (0.25)	2.1 (0.61)	2.0 (0.74)	1.6 (0.65)
**CSF α-syn SAA positivity**	0 (0%)	106 (100%)	0 (0%)	98 (100%)	204 (23.1%)

Data are shown as mean (s.d.) unless otherwise specified.

^a^Based on whether the participant had been told that he/she seems to ‘act out his/her dreams’ (single-question screen for REM sleep behavior disorder^39^).

^b^Data were only available for BioFINDER-2.

^c^This diagnostic entity consisted of a behavioral variant of FTD, unspecified subtype of FTD, non-fluent variant of primary progressive aphasia, semantic variant of primary progressive aphasia, corticobasal syndrome and progressive supranuclear palsy.

^d^The majority of these participants were diagnosed at follow-up visits (Extended Data Table 1).

^e^Measured in a temporal meta-ROI^34^.

***The proportion of participants clinically diagnosed with DLB, PD or PDD in the AD−/LB+ group was significantly higher than in the AD+/LB+ group (chi-squared test, *P* < 0.001). MMSE, mini-mental state examination; VaD, vascular dementia; FTD, frontotemporal dementia; SUVR, standardized uptake value ratio; ROI, region of interest.

### Cross-sectional associations with clinical outcomes

**Fig. 2:**
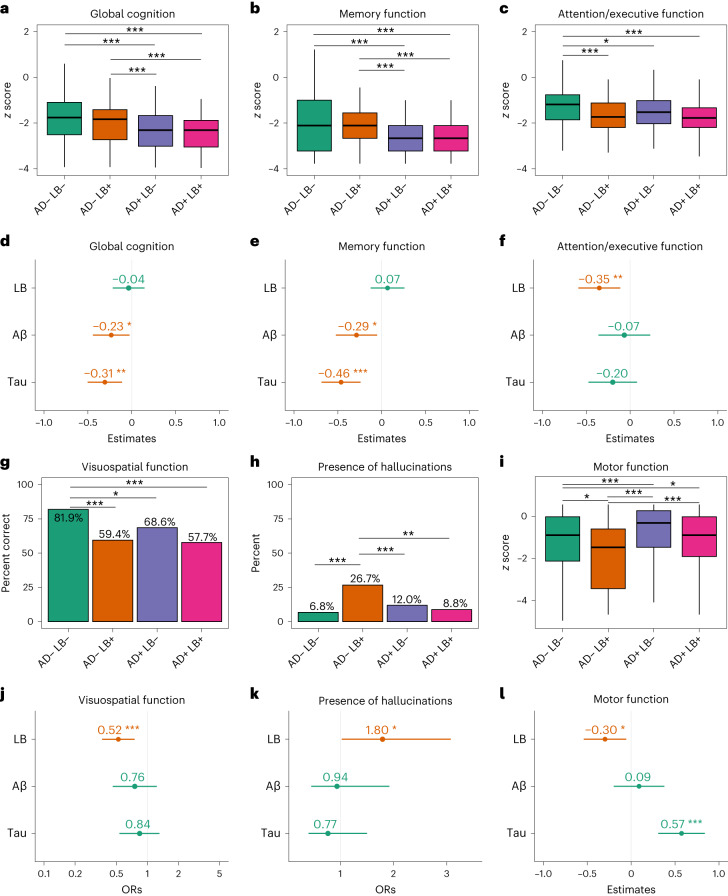
Comparisons between AD/LB groups and the independent effects of LB, Aβ and tau pathologies on clinical outcomes. **a**–**l**, Analyses were performed using linear regression models with AD/LB groups (**a**–**c**,**g**–**i**) or all three binarized pathologies (**d**–**f**,**j**–**l**) as independent variables in the same model, adjusted for age, sex, education (for cognitive outcomes) and cognitive stage (MCI/dementia). In **g**,**h**,**j**,**k**, logistic regression models with the same covariates were used because the outcomes were binary. Outcomes were *z* scored (according to the distribution in Aβ-negative controls) cognitive tests (**a**–**f**) and motor questionnaires (**i**,**l**) or binary assessment of correct visuospatial task (**g**,**j**) or presence of hallucinations (**h**,**k**). Boxes (**a**–**c**,**g**–**i**) show interquartile range, the horizontal lines are medians and the whiskers were plotted using the Tukey method. In **d**–**f**,**l**, the dot/center shows the estimate of the pathology and the error bars show the 95% CI, where negative values equal worse performance. In **j**,**k**, the dot/center represents ORs, where values <1 equal a decrease, and error bars show the 95% CI of the ORs. Worse performance is marked in red. AD positivity was defined as the presence of both Aβ and T. LB positivity was defined as the presence of an abnormal α-syn SAA result. The effect of LB pathology on clinical outcome with/without adjusting for Aβ and T is shown in Extended Data Table 2. Overall, 302 participants were AD−/LB−, 106 were AD−/LB+, 377 were AD+/LB− and 98 were AD+/LB+ and 204 were LB+, 607 were Aβ+ and 489 were tau+. The statistical analyses with corrections for multiple comparisons are shown in Supplementary Fig. 1. Missing data are shown in Supplementary Table 1. *P < 0.05; **P < 0.01; ***P < 0.001 (two-sided).

Next, we studied the effects of the different key brain pathological changes on clinical deficits at baseline. When comparing group differences (Fig. 2a–c,g–i), patients who were AD−/LB+, AD+/LB− or AD+/LB+ had worse attention/executive and visuospatial function than patients who were AD−/LB−. Patients who were AD+/LB− and AD+/LB+ had worse memory and global cognition compared to patients who were either AD−/LB− or AD−/LB+. Patients who were AD−/LB+ had worse motor function and exhibited a higher prevalence of hallucinations compared to all the other groups.

Multivariable linear regression analyses using the measures of LB, Aβ and tau pathologies as predictors in the same model and the clinical symptoms as outcomes could overall confirm the cognitive and clinical profiles from the group analyses (Fig. 2d–f,j–l). When used simultaneously as predictors, LB pathology, but not Aβ nor tau pathologies, had independent effects on worse attention/executive, visuospatial and motor function and was associated with higher prevalence of hallucinations. Aβ and tau, on the other hand, had independent effects on worse memory and global cognition. Model specifications of the effects of LB with/without adjusting for Aβ and tau pathology are shown in Extended Data Table 2. Finally, we found no association between presence of LB and Aβ status (*P* = 0.14, adjusted for age) or LB and tau status (*P* = 0.065, adjusted for age).

### Longitudinal associations with cognitive outcomes

**Fig. 3:**
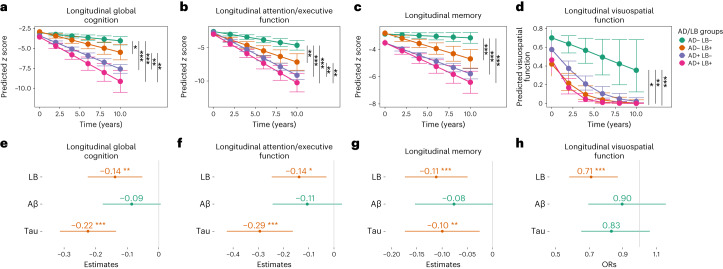
Comparisons between AD/LB groups and the independent effects of LB, Aβ and tau pathologies on longitudinal cognitive function. **a**–**h**, Significant effects (two-sided) were examined using linear mixed-effects (LME) models. The AD/LB group × time interaction was examined, adjusted for age, sex, education and cognitive stage (MCI/dementia) (**a**–**d**). The interaction time × all three pathologies (binarized) were examined in the same LME model adjusted for age, sex and education, to examine the independent effect of pathology and cognitive progression (**e**–**h**). Outcomes were *z* scored cognitive tests according to the distribution in Aβ-negative controls. The effect of LB pathology on clinical outcome with/without adjusting for Aβ and tau is shown in Extended Data Table 3. Estimated marginal means and 95% CI of the means obtained from LME models by AD/LB group (**a**–**d**). The dot/center shows the interaction estimates of time × pathology and error bars represent the 95% CI (**e**–**h**). Binomial mixed-effects models were used as the outcome was binary (**c**,**g**). Overall, 302 participants were AD−/LB−, 106 were AD−/LB+, 377 were AD+/LB− and 98 were AD+/LB+ and 204 were LB+, 607 were Aβ+ and 489 were tau+. The statistical analyses with corrections for multiple comparisons are shown in Supplementary Fig. 2. Missing data are shown in Supplementary Table 2. *P < 0.05; **P < 0.01; ***P < 0.001 (two-sided).

Next, we studied the effects of the different brain pathologies on longitudinal changes in cognitive function. First, we examined the effects of the AD/LB group classification on longitudinal cognitive function using linear mixed-effects models. We found that patients who were AD−/LB+, AD+/LB− or AD+/LB+ progressed faster in all cognitive outcomes than those who were AD−/LB− (Fig. 3a–d). Deterioration in global cognitive function and attention/executive function was seen in both AD+ and LB+ groups, but those with AD pathology declined even faster (Fig. 3a,b). To make sure that differences in trajectories were not driven by baseline group differences, we performed a sensitivity analysis using change in cognition from baseline as outcome and adjusted the model for baseline cognitive score, which confirmed the significant differences (Extended Data Table 3). Second, when studying the independent effects of LB, Aβ and tau pathologies, we found that only LB pathology was independently associated with a faster decline in visuospatial function (Fig. 3h). Further, both LB and tau pathologies were independently associated with a faster decline in memory, attention/executive and global cognitive function (Fig. 3e–g). Model details with/without adjusting for Aβ and tau are shown in Extended Data Table 4. To examine whether LB pathology provided prognostic information in addition to identifying the clinical features of DLB, PD or PD with dementia (PDD), we adjusted these models for a clinical baseline diagnosis of DLB/PD/PDD. We found that LB pathology was still an independently significant predictor of cognitive decline in all cognitive domains (Extended Data Table 5).

## Discussion

Clinicopathological postmortem association studies suggest that both AD and LB pathologies play a substantial role in determining the clinical phenotype in patients where the two pathologies coexist^18–20^; however, neuropathological evaluation only provides a terminal end-stage window of the co-occurring pathologies, which is difficult to correlate with the earlier signs and the clinical trajectories of patients assessed longitudinally for many years. Before the introduction of α-syn SAAs, the lack of a reliable biomarker for LB pathology hampered the design of clinicopathological association studies in vivo. Here we provide a combined in vivo evaluation of biomarkers highly specific for AD and LB pathology and their correlation with the clinical phenotype in a large heterogeneous cohort of patients with cognitive impairment. The results show that LB pathology had effects on the clinical profile independent of Aβ and tau pathologies with cross-sectional impairment in attention/executive, visuospatial and motor functions. LB pathology also had independent associations with a faster decline over time in global, attention/executive, memory and visuospatial functions. Overall, adding classification by LB status to the standard AD-centered Aβ/tau classification identifies patient subgroups with distinct cognitive profiles and clinical trajectories. The cognitive findings from this in vivo biomarker-based patient classification are in line with those obtained in neuropathologically verified cohorts with cognitive data showing an association between LB pathology and antemortem deficits in attention, executive and visuospatial functions also in participants with mixed AD and LB pathology^21–24^.

In the present study, we show that this biomarker-based classification cannot be replaced by clinical phenotyping as only 21% at baseline and 32% during follow-up of the patients who were LB+ fulfilled the clinical criteria for PD or DLB (Table 1). This can be compared to patients who are AD+, where 50% had a clinical baseline diagnosis of AD and 87% during follow-up (note that if a patient fulfilled the criteria for both AD and DLB this was coded as DLB). This was specifically highlighted when we adjusted the models for a clinical DLB/PD/PDD diagnosis at baseline and found that LB pathology was still a significant predictor of cognitive decline (Extended Data Table 5). In particular, the presence of both pathologies (AD+/LB+) seems more difficult to identify clinically as only 9% in this group fulfilled the criteria for DLB/PD at baseline (16% during follow-up) versus 31% (46% during follow-up) in the AD−/LB+ group (*P* < 0.001; Table 1). This may be related to a significantly lower frequency of motor symptoms and hallucinations in the AD+/LB+ group compared to the AD−/LB+ group (i.e., fewer core clinical features of PD^15^ or DLB;^17^ Fig. 2h,i). The demonstration of concurrent AD and LB pathologies has previously mainly been possible postmortem by neuropathologic studies, which is of little help to the patients, yet these patients are particularly important to identify in the clinic because of the fast clinical progression (Fig. 3a–d). Moreover, the exclusion or the separate evaluation of patients with these profiles in trials assessing the effect of new therapies for AD will be essential for determining the impact of disease-modifying AD treatments without the confounding effect of a concomitant LB pathology driving disease progression independently and despite removal of AD pathology.

Both for trial screenings and clinical practice, it would be advantageous to have several options for analyzing LB pathology, depending on the setting and available instruments. We used CSF for the α-syn SAA, which requires a lumbar puncture, but several studies successfully used skin biopsies instead^25–27^. An even less-invasive approach would be to measure α-syn seeding activity in blood, which is currently under development with promising preliminary results^28^.

In this study, 21% of patients who were AD+ showed evidence of LB pathology, which is lower than the percentage of concomitant LB pathology detected by neuropathological postmortem studies (33–42%) (refs. ^3,29^). One explanation could be the effect of sample selection. In the BioFINDER cohorts, patients are consecutively recruited based on referrals to secondary memory clinics, whereas those who undergo neuropathological examination are often more selected^28^. Another possibility is that this is related to the sensitivity of α-syn SAAs compared to neuropathological verification of LB pathology. α-Syn SAAs have a very high sensitivity for the transitional limbic and diffuse neocortical stages of LB pathology, but lower for focal types of LB pathology limited to the amygdala or possibly the brainstem^8,9,11,14^. Notably, the amygdala-only variant contributes a substantial proportion of AD cases with α-syn pathology in postmortem studies and would thus go largely unnoticed using α-syn SAAs^30^; however, the amygdala-predominant variant does not lead to a clinical LBD presentation and is less clinically relevant^31^.

Regarding the prevalence of LB and AD pathologies in relation to increasing age, this was more prominent for Aβ and tau, than LB. Further, after the age of 80 years, the prevalence of Aβ and tau did not seem to further increase in our memory clinic population (Fig. 1d). The results of a large neuropathological study support our finding of a lack of, or only subtle, association between age and LB pathology in cognitively impaired individuals^32^. The seemingly paradoxical finding that LB pathology becomes more prevalent with age on a population basis but less so in cognitively impaired/memory clinic samples^32^, might be explained by the increasing prevalence of other pathologies (for example limbic-predominant age-related TDP-43 encephalopathy (LATE) and vascular lesions) contributing to cognitive decline, resulting in a lack of clear increase in the proportion of LB pathology among memory clinic patients^33^.

A limitation of the present study concerns the lack of longitudinal analyses of non-cognitive symptoms, including structured assessments of fluctuations in alertness/attention and signs of REM sleep behavioral disorders, often seen in individuals with LB pathology^17^. Future studies should examine the longitudinal changes of all LB-related symptoms (not just cognition) in more detail to better define the clinical trajectory of patients with coexisting AD and LB pathology. It would be of particular interest to see whether or when the AD+/LB+ group would develop motor dysfunction similarly to the AD−/LB+ group. As for the measure of motor dysfunction in the statistical analysis, we used an informant-based questionnaire, but data from a formal objective assessment (for example UPDRS-III) were lacking in this study; however, we still identified a significant association between motor function and LB pathology (Fig. 2i,l) indicating the adequate validity of the questionnaire. Moreover, due to the study designs, AD biomarkers for tau pathology differed between patients in BioFINDER-2 and BioFINDER-1; however, the CSF assay (Lilly-developed CSF P-tau217) used for tau in BioFINDER-1 has shown a very high agreement with tau-PET status (used in BioFINDER-2) (ref. ^34^).

This study has highlighted the potential improvement of including an LB biomarker in the clinical assessment of a patient with cognitive impairment, with substantial diagnostic and prognostic implications. Still, it is important to remember that other pathologies currently lack corresponding in vivo biomarkers, such as LATE and four repeat (4R) tauopathies (such as progressive supranuclear palsy and corticobasal degeneration)^35,36^. Although non-AD and non-LB pathologies may be less common, occur more often in older people or have less prominent clinical impact^22,29,37,38^, it is crucial with continued efforts to develop corresponding biomarkers to implement a more complete, pathology-based precision-medicine approach.

In summary, in this large heterogeneous sample of patients with cognitive impairment referred to secondary memory clinics, our results indicate that classifying patients based on the presence of LB, Aβ and tau pathologies identifies subgroups with distinct clinical phenotypes and trajectories that are not captured by using clinical syndromes or classification according to the standard Aβ and tau biomarkers. Analyzing LB pathology in vivo could provide a better precision-medicine approach for the clinical management of patients with cognitive impairment and for designing drug trials of participants with AD and LBD.

## Methods

### Participants and clinical diagnostics

All participants were part of the BioFINDER-1 (NCT01208675; *n* = 398) or BioFINDER-2 (NCT03174938; *n* = 485) studies, described previously^34,40,41^. The inclusion criteria were (1) referred to participating secondary memory clinics due to cognitive symptoms recognized by the patient, caregiver and/or the referring physician; (2) age 40–100 years; and (3) speaks and understands Swedish to the extent that an interpreter is not necessary for the patient to fully understand the study information and cognitive tests. The exclusion criteria were (1) significant unstable systemic illness or organ failure, such as terminal cancer, which makes it difficult to participate in the study; and (2) current significant alcohol or substance misuse. Only participants (1) with MCI or dementia at baseline; (2) with complete biomarker data for Aβ, tau and α-syn (LB pathology); and (3) referred to the participating memory clinics of Skåne University Hospital or the hospital of Ängelholm in Sweden due to cognitive symptoms recognized by the patient, caregiver and/or the referring physician, were included in the present study. All patients were enrolled and underwent baseline examination from 2007 to 2015 (BioFINDER-1) or from 2017 to 2021 (BioFINDER-2). MCI was classified as not fulfilling the criteria for dementia (major neurocognitive disorder according to DSM-5 (ref. ^42^)) and performing worse than −1.5 × s.d. in at least one of the cognitive domains of memory, attention/executive, verbal or visuospatial function. In BioFINDER-1, this was assessed by a senior neuropsychologist after a thorough neuropsychological battery, as described in detail previously^43^. In BioFINDER-2, the MCI classification was operationalized as performing worse than −1.5 *z* scores in any cognitive domain according to regression-based norms accounting for age and education and the test performance in Aβ-negative controls^44^ (see elsewhere^45,46^ for a description on the regression-based *z* scores). Cognitive domain *z* scores were derived by calculating the mean *z* score of the tests in each of the following domains: attention/executive function (trail-making test A, trail-making test B and symbol digit modalities test), verbal ability (verbal fluency animals and the 15-word short version of the Boston naming test), memory (ten-word delayed recall from the Alzheimer’s disease assessment scale (ADAS)) and visuospatial function (incomplete letters and cube analysis from the visual object and space perception battery). Dementia was classified according the DSM-5 criteria for major neurocognitive disorders^42^.

A clinical diagnosis of AD was based on the DSM-5 criteria for mild or major neurocognitive disorder due to AD^42^. In addition, signs of Aβ positivity were required in agreement with the National Institute of Aging-Alzheimer’s Association^13^ and International Working Group^4^ criteria for AD. The biomarker for Aβ positivity was not the same as the Elecsys CSF Aβ42/Aβ40 assay used in the present study (described in detail previously^41^), which explains why some with a clinical AD diagnosis (Table 1 and Extended Data Table 1) are AD− in the AD/LB biomarker classification. Note that this clinical diagnosis was only used for describing the sample (Table 1 and Extended Data Table 1). The purely biomarker-driven classification for AD pathology (Aβ and tau positivity, regardless of clinical syndrome) was used in the statistical analysis. A clinical diagnosis of DLB was based on the McKeith criteria for probable DLB (MCI^16^ or dementia^17^ stage) and PD was based on previously published criteria for PD and PDD^47^ (where the DSM-5 criteria were used to identify presence of dementia/major neurocognitive disorder^42^). The PD diagnoses are detailed in Supplementary Table 3, where the criteria used in the present study (Gelb et al.^47^) are compared to the diagnostic classification according to the Movement Disorder Society (MDS) criteria^15^. If a participant fulfilled the criteria for both AD and DLB/PD he/she was coded as DLB or PD in the analyses. The other diagnostic entities were diagnosed according to published criteria^42,48–51^, as previously described^34^. Notably, to increase the clinical diagnostic accuracy, the diagnoses were also determined during a longitudinal follow-up of symptoms and advanced investigations at a secondary memory clinic (Extended Data Table 1). Note that α-syn SAA results were not available when the diagnoses were determined.

All participants or their legal representatives provided written informed consent. Ethical approval was given by the Regional Ethical Committee in Lund, Sweden.

### Clinical outcomes

All clinical outcomes, except the presence of hallucinations and correctly completed visuospatial task, were *z* scored according to distribution in Aβ− cognitively unimpaired participants in BioFINDER-1 and BioFINDER-2. The modified Preclinical Alzheimer Cognitive Composite-5 (mPACC5; also referred to as PACC) was used as a measure of global cognition, containing tests of memory, executive, attention and verbal function^52^. It was calculated based on the previously described PACC5, using the MMSE, symbol digit modality test (SDMT) and animal fluency^52^. As the memory tests logical memory and the free and cued selective reminding tests were not available in BioFINDER, the ten-word delayed recall task from ADAS-cognition (ADAS-cog)^53^ was used (weighted twice), as previously applied in several studies^54,55^. The mPACC5 was thus calculated using *z* scores based on the distribution in Aβ− cognitively unimpaired in the following way: (MMSE + (ADAS-cog delayed recall × 2) + SDMT + animal fluency) / 5. Memory was measured using the ten-word delayed recall task from ADAS-cog^53^. Attention/executive function was measured using the SDMT^56^. If SDMT was not available, the serial 7s task of the MMSE was used (Supplementary Tables 1 and 2 detail missingness)^57^. Visuospatial function was measured using the incomplete letters task from the visual object and space perception battery and if incomplete letters was not available (per study design only available in BioFINDER-2), the pentagon copying task from the MMSE was used. As pentagon copying is scored as either normal (1) or abnormal (0), the incomplete letters task was binarized at ≤18 of 20 points (mean − 2 × s.d. in Aβ-negative cognitively unimpaired participants in BioFINDER-2).

Presence of hallucinations (yes / no) was assessed using the informant-based cognitive impairment questionnaire (CIMP-QUEST)^58^, item eight in the associated symptom’s part (‘Does the patient has hallucinations, seeing, hearing or feeling things that don’t actually exist but that he/she has a clear experience of?’). Motor function was measured using the combined score from all motor symptoms in CIMP-QUEST, covering aspects of bradykinesia, reaction, changed the way of walking, poorer balance, clumsier hands, changed facial expressions and dysarthria.

### Biomarker of Aβ

The CSF Aβ42/Aβ40 ratio was used to define Aβ positivity, as Aβ-PET was not included at baseline in BioFINDER-1 and was not available in BioFINDER-2 participants with dementia, per study design. Aβ42 and Aβ40 were analyzed on a cobas e 601 analyzer using the Roche NeuroToolKit. The threshold for positivity was defined using mixture modeling statistics^59^. In BioFINDER-1, the previously established cutoff of CSF Aβ42/Aβ40 < 0.066 was used^60^. For BioFINDER-2, the cutoff was established in all BioFINDER-2 participants (cognitively unimpaired and impaired) with available CSF Aβ42 and Aβ40 data (*n* = 1,076), resulting in a cutoff of <0.080 (the difference in cutoffs between cohorts is explained by preanalytical differences in that LoBind tubes were used in BioFINDER-2 but not in BioFINDER-1 (refs. ^61,62^) for preanalytical protocols and differences).

### Biomarker of tau

Tau positivity was defined as either abnormal CSF P-tau217 (BioFINDER-1) or abnormal tau-PET (BioFINDER-2). CSF P-tau217 was measured using the Meso Scale Discovery platform using an assay developed by Eli Lilly and tau-PET was performed using RO948 labeled with radioactive fluorine [18F], as previously described^34^. SUVR was measured in a temporal meta-ROI using the inferior cerebellar cortex as reference region^34^. Cutoffs were established at the mean + 2 × s.d. in Aβ− controls as previously described and the cutoff for CSF P-tau217 was >11.42 pg ml^−1^ and for tau-PET > 1.32 SUVR (ref. ^34^).

### Preparation of recombinant α-synuclein (LB pathology)

The purification of recombinant wild-type α-syn was performed as previously reported^8^, with minor modifications. Briefly, transformed *Escherichia* *coli* BL21 (DE3) bacteria (New England Biolabs) from a glycerol stock were streaked on a selective plate (Kan+, 50 µg ml^−1^, kanamycin from Sigma) and incubated at 37 °C overnight. A single colony was selected and inoculated into 5 ml Luria Broth (Sigma) with kanamycin and allowed to grow for 4–5 h at 37 °C with continuous agitation at 250 r.p.m. This starter culture was then added to 1 l Luria Broth containing kanamycin and the overnight express auto-induction system (Merk-Millipore 71300-4) in a fully baffled flask. Cells were grown in a shaking incubator at 37 °C, 200 r.p.m. overnight. The next day, the culture was split into four 250-ml flasks and centrifuged at 3,200*g* for 10 min at 4 °C. The pellet was gently resuspended in 25 ml osmotic shock buffer containing 40% sucrose (Sigma), 2 mM EDTA (Sigma) and 30 mM Tris (Bio-Rad) at pH 7.2 using a serological pipette and incubated for 10 min at room temperature under mild agitation on a rotator mixer. The solution was then centrifuged at 9,000*g*, 20 min at 20 °C and each pellet was resuspended in 10 ml ice-cold Milli-Q water. The four suspensions were pooled into two 50-ml tubes and 20 µl of saturated MgCl_2_ (Sigma). After 3 min incubation under mild rocking on ice, the suspension was centrifuged at 9,000*g* for 30 min at 4 °C and the supernatant was collected into a 100-ml glass beaker. The pH was reduced to pH 3.5 by adding 400–600 µl HCl 1 M (PanReac AppliChem) and incubated under stirring for 10 min at room temperature. After a second centrifugation at 9,000*g* for 30 min at 4 °C, the supernatant was collected into a clean 100-ml glass beaker. The pH was adjusted to 7.5 by adding 400–600 µl NaOH 1 M (Sigma). The protein extract was filtered through a 0.22-µm filter (Merk-Millipore), loaded into a Ni–NTA column (Cytiva 17525501) on an NGC chromatography system (Bio-Rad) and washed with 20 mM Tris, pH 7.5 at room temperature. The column was further washed with 50 mM imidazole (Sigma) in Tris 20 mM, pH 7.5, generating a peak that was not collected. A linear gradient up to 500 mM imidazole in 20 mM Tris, pH 7.5 was performed and the peak was collected between 30% and 75% of imidazole buffer (150 and 375 mM, respectively). This peak was loaded onto an anion exchange column Q-HP (Cytiva 17115401) and washed in Tris 20 mM, pH 7.5, followed by another washing in 100 mM NaCl in Tris 20 mM, pH 7.5. Again, a linear gradient up to 500 mM of NaCl in Tris 20 mM pH 7.5 was carried out to collect the peak between 300 and 350 mM NaCl. The fractions were pooled and filtered through a 0.22-µm filter and dialyzed against Milli-Q water overnight at 4 °C using a 3.5 kDa MWCO dialysis membrane (Thermo-Scientific). The next day, the protein was moved into fresh Milli-Q water and dialyzed for 4 h more. The protein concentration was measured by a spectrophotometer using a theoretical extinction coefficient at 280 nm of 0.36 (mg ml^−1^) − 1 cm^−1^. Finally, the protein was lyophilized for 6 h and stored in aliquots at a final concentration of 1 mg ml^−1^ after resuspension into 500 µl phosphate buffer (PB; Sigma) 40 mM, pH 8.0. Lyophilized aliquots were stored at − 80 °C until usage.

### α-Syn RT-QuIC analyses

α-Syn RT-QuIC analyses were performed blinded to clinical status and diagnosis of the participant and according to an established protocol^8,63,64^, with minor modifications. Briefly, six 0.8-mm silica beads (OPS Diagnostics) per well were pre-loaded into black 96-well plates with a clear bottom (Nalgene Nunc International). CSF samples were thawed and vortexed for 10 s before use. Then, 15 μl CSF was added to a 85 μl reaction mix composed of 40 mM PB, pH 8.0, 170 mM NaCl, 10 mM thioflavin-T (Sigma), 0.0015% SDS (Bio-Rad) and 0.1 g l^−1^ of filtered recombinant α-syn (100-kDa Amicon centrifugal filters, Merck Millipore). Plates were closed with a plate sealer film (Nalgene Nunc International) and incubated into a FLUOstar Omega plate reader (BMG Labtech) at 42 °C with intermittent double orbital shaking at 400 r.p.m. for 1 min, followed by 1 min rest. Fluorescence was measured every 45 min with 450 nm excitation and 480 nm emission filters during the 30 h test run. Samples and controls were run in quadruplicate and considered positive after the first run when at least three out of four replicates reached a threshold arbitrarily set at 30% of the median of Imax values reached by the positive control replicates. To keep the risk of false-positive results at a minimum, we repeated three times the analysis of samples showing seeding activity in only one or two out of four replicates in the first run. We considered a positive result only when at least 4 of the 12 total replicates reached the threshold. We used 30 different batches of α-syn recombinant protein throughout the study. Each batch underwent a quality control test before use. We ran at least one positive and negative control on each plate. The positive controls were chosen from patients with probable or definite DLB or PD whose CSF samples gave four out of four positive replicates during screening. In each validated experiment (plate) included in the final analysis, the positive controls showed at least three out of four positive replicates.

### Statistical analyses

In cross-sectional analyses, the AD/LB group, age, sex (assigned, not self-reported), cognitive stage (MCI or dementia) and, for cognitive test outcomes, years of education, were used as independent variables in general linear regression models. Dependent variables were either cognitive function, motor function or presence of hallucinations. Next, binarized Aβ, tau and LB pathology (to facilitate easier comparison of estimates), were used instead of the AD/LB group. Logistic regression models were used for the binary outcomes (visuospatial function and presence of hallucinations). In longitudinal analyses, LME models were used (R packages lme4 and the function lmer for continuous outcomes and glmer for binarized outcomes were used and *P* values were calculated using the package lmerTest). Cognitive function was used as an outcome and significant results were presented for the AD/LB group × time and pathology × time interactions. The models also included age, sex, cognitive stage (MCI or dementia), years of education and random slopes and intercepts. For models including pathology × time, the interaction between time and all covariates were also included. In a sensitivity analysis, the LME models using AD/LB group × time were also adjusted for baseline cognitive test result (if there was a difference between AD/LB groups at baseline) with change from baseline in cognitive test result as outcome. All available data were used in the statistical analyses. Missing data and number of participants at each visit are described in Supplementary Tables 1 and 2. A two-sided *P* value <0.05 was considered to indicate statistical significance. Multiple comparison corrections were performed using the false discovery rate method at *α* = 0.05, applying correction per outcome (six comparisons for AD/LB group comparisons and three for the independent effects of LB, Aβ and tau pathology). The statistical analyses were performed using R v.4.1.

### Reporting summary

Further information on research design is available in the Nature Portfolio Reporting Summary linked to this article.

## Online content

Any methods, additional references, Nature Portfolio reporting summaries, source data, extended data, supplementary information, acknowledgements, peer review information; details of author contributions and competing interests; and statements of data and code availability are available at 10.1038/s41591-023-02449-7.

### Supplementary information

Supplementary Information Supplementary Figs. 1 and 2 and Supplementary Tables 1–3.

Reporting Summary

## Figures and Tables

**Extended Data Table 1:**
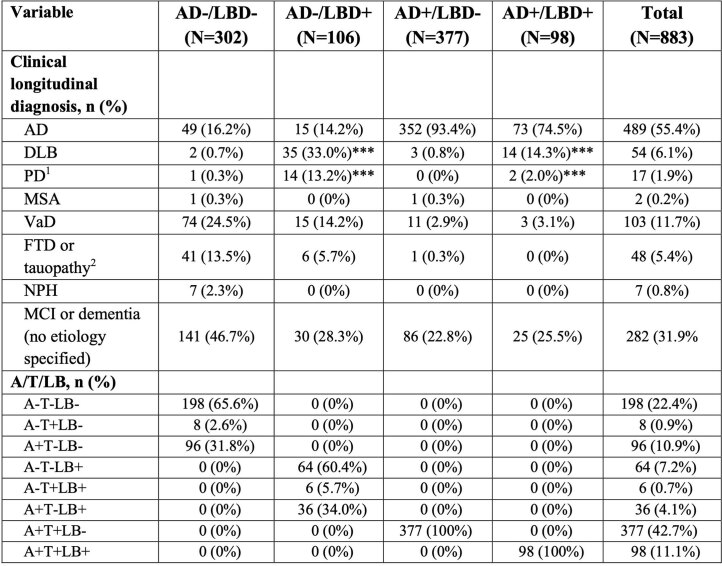
Further characteristics of the AD/LB groups

^1^A detailed classification of the PD diagnosis is provided in Supplementary Table 3.

^2^This diagnostic entity comprised of behavioral variant of FTD, unspecified subtype of FTD, non-fluent variant of primary progressive aphasia, semantic variant of primary progressive aphasia, corticobasal syndrome, and progressive supranuclear palsy.

*** The proportion of participants clinically diagnosed with DLB, PD or PDD in the AD-/LB+ group was significantly higher than in the AD+/LB+ group (Chi^2^ test p < 0.001).

**Extended Data Table 2:**
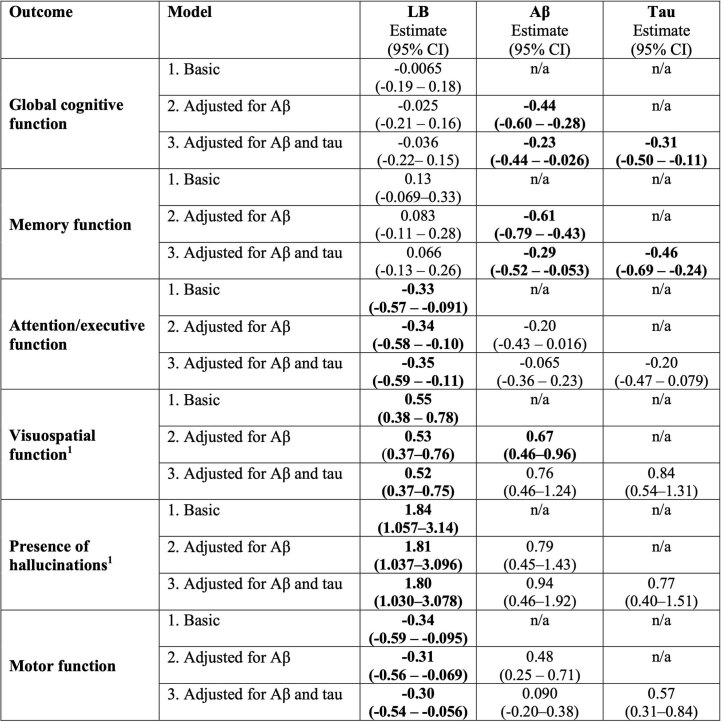
Cross-sectional association between pathology and baseline clinical outcome

Linear regression models were used for continuous outcomes (global, memory, attention/executive, and motor function) with results presented as betas. Logistic regression models were used for binary outcomes (visuospatial function and presence of hallucinations) with results presented odds ratios (OR). Basic models included LB status, age, sex, cognitive status (MCI or dementia) and years of education (education was not included in models with motor function or hallucinations) as predictors. At the second step, Aβ was added to the model and at the third step tau. The third model is thus the same as shown in Fig. 2d–f and j–l. Estimates in bold indicate a significant worse effect on the outcome. Note that LB status refers to the result of the α-syn SAA (positive/negative).

n/a, not applicable (not included in model).

^1^Data presented as ORs (logistic regression models since the outcome was binary).

**Extended Data Table 3:**
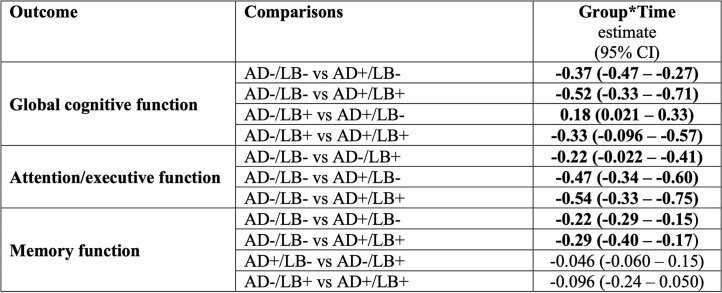
AD/LB group comparisons of change in cognition adjusted for baseline cognition

Linear mixed-effects (LME) models examining the interaction between AD/LB group and time (years) using change in cognition (global, memory or attention/executive) since baseline as outcome. The models were adjusted for baseline cognition (global, memory or attention/executive z-score), age, sex, cognitive stage (MCI or dementia), years of education, and included a random slope and intercept. The sensitivity analyses were performed in all instances where a baseline group difference was found (Fig. 2a–c). Change in visuospatial function was not examined in the sensitivity analysis since it was a binary variable (0 or 1) and not a continuous variable as the other cognitive variables.

**Extended Data Table 4:**
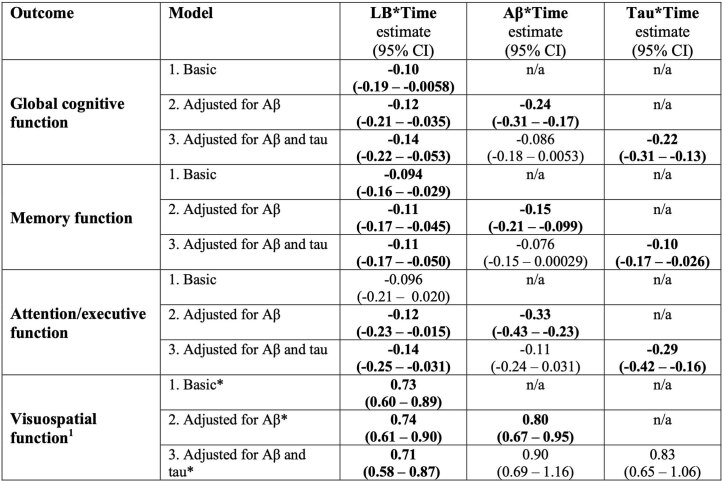
Longitudinal association between pathology and clinical outcome

Linear mixed-effects (LME) models examining the interaction between pathology and time (years) using cognition as outcome. Basic models included the interaction between time and α-syn, age, sex, cognitive stage (MCI or dementia) and years of education, and included a random slope and intercept At the second step, Aβ was added to the model and at the third step tau. The third model is thus the same as shown in Fig. 3e–h. Note that LB refers to a positive α-syn SAA biomarker.

^1^ORs are presented for visuospatial function since it is a binary outcome.

**Extended Data Table 5:**
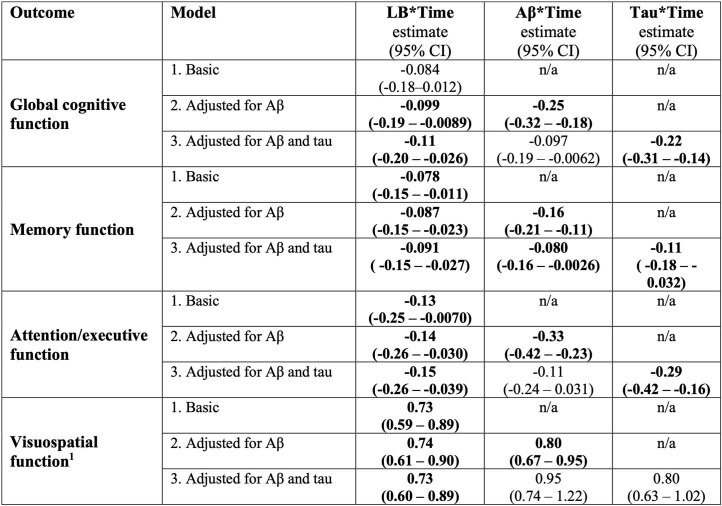
Longitudinal association between pathology and clinical outcome adjusted for a clinical baseline diagnosis of DLB or PD/PDD

Linear mixed-effects (LME) models examining the interaction between pathology and time (years) using cognition as outcome. Basic models included the interaction between time and α-syn, a clinical diagnosis of DLB/PD/PDD, age, sex, cognitive stage (MCI or dementia) and years of education. At the second step, Aβ was added to the model and at the third step tau. Note that LB refers to a positive α-syn SAA biomarker.

^1^ORs are presented for visuospatial function since it is a binary outcome.

## Data Availability

Anonymized data will be shared by request from a qualified academic investigator for the sole purpose of replicating procedures and results presented in the article and as long as a data transfer is in agreement with EU legislation on the General Data Protection Regulation and decisions by the Ethical Review Board of Sweden and Region Skåne, which should be regulated in a material transfer agreement.

## Code availability

No custom code or mathematical algorithm that was central to the conclusions was used in this study.
