# Supplementary material for: Clinical effects of Lewy body pathology in cognitively impaired individuals
Source: Nat Med. 2023 Jul 18;29(8):1964–70. doi: 10.1038/s41591-023-02449-7 (PMC10427416; doi:10.1038/s41591-023-02449-7)
Supplement: Supplementary file 1 — Supplementary Figs. 1 and 2 and Supplementary Tables 1–3. [file 41591_2023_2449_MOESM1_ESM.pdf]

nature medicine

Article

https://doi.org/10.1038/s41591-023-02449-7

Clinical effects of Lewy body pathology in cognitively impaired individuals

In the format provided by the authors and unedited

ONLINE SUPPLEMENT

SUPPLEMENTARY FIGURES

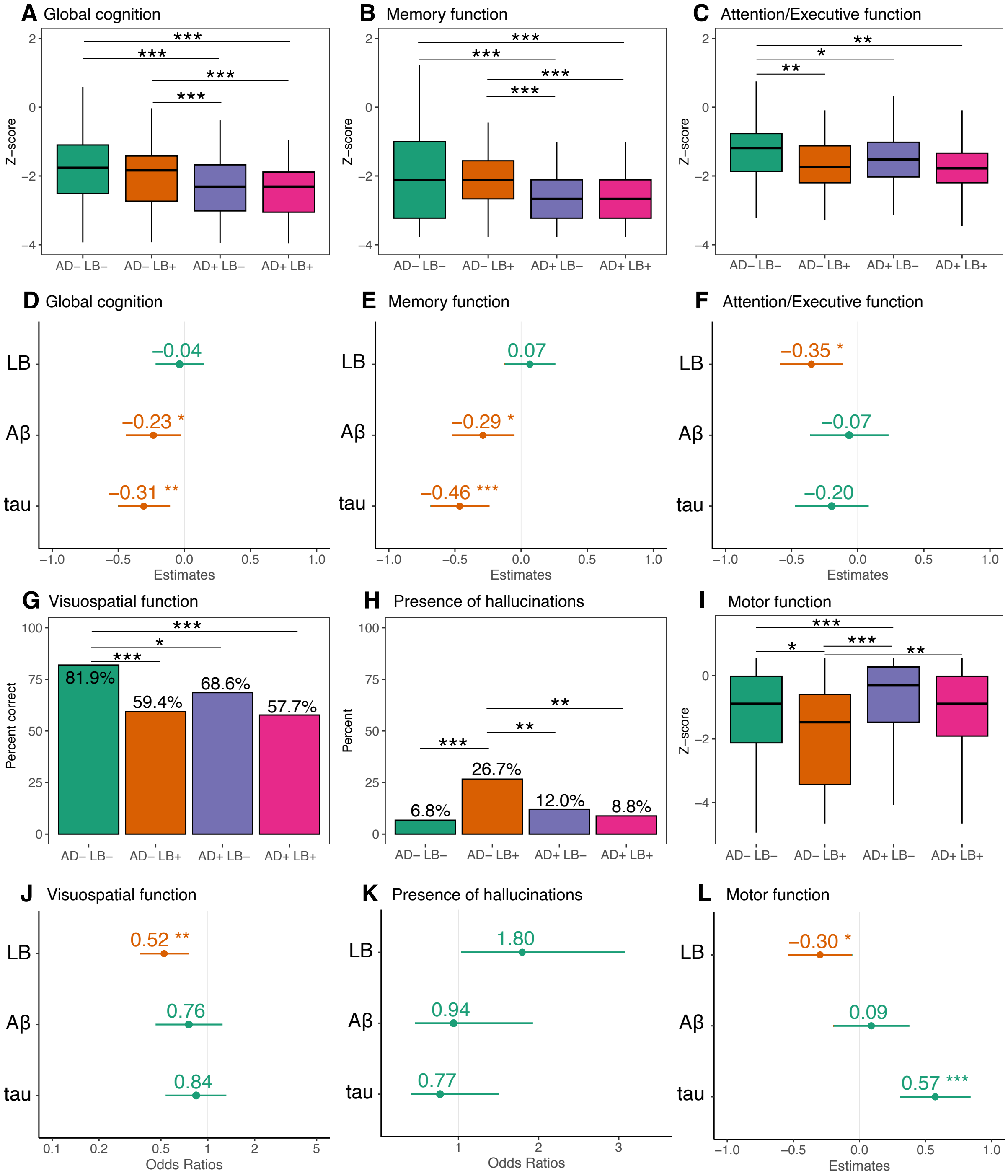

Supplementary Figure 1A-L. Comparisons between AD/LB groups and the independent effects of LB, Aβ and tau pathologies on clinical outcomes with corrections for multiple comparisons. The analyses were performed using linear regression models with AD/LB groups (A-C, G-I) or all three binarized pathologies (D-F, J-L) as independent variables in the same model, adjusted for age, sex, education (for cognitive outcomes), and cognitive stage (MCI/dementia). In G, H, J, and K, logistic regression models with the same covariates were used since the outcomes were binary. Outcomes were z-scored (according to the distribution in Aβ-negative controls) cognitive tests (A-F)

and motor questionnaires (I, L) or binary assessment of correct visuospatial task (G, J) or presence of hallucinations (H, K). Boxes (A-C, G-I) show interquartile range, the horizontal lines are medians and the whiskers were plotted using the Tukey method. In D-F, and L, the dot/center shows the estimate of the pathology and the error bars the 95% confidence interval, where negative values equal worse performance. In J and K, the dot/center represents odds ratios where values below 1 equals a decrease, and the error bars the 95% CI of the odds ratios. Worse performance is marked in red. AD positivity was defined as presence of both Aβ and tau. LB positivity was defined as presence of abnormal α-syn SAA result. 302 participants were AD-/LB-, 106 AD-/LB+, 377 AD+/LB-, and 98 AD+/LB+. 204 were LB+, 607 Aβ+, and 489 tau+. Multiple comparison corrections were applied per outcome using the FDR method (at α=0.05). Missing data are shown in Supplementary Table 1.
* $p<0.05$; ** $p<0.01$; *** $p<0.001$

Abbreviations: Aβ, beta-amyloid; AD, Alzheimer's disease. LB, Lewy body

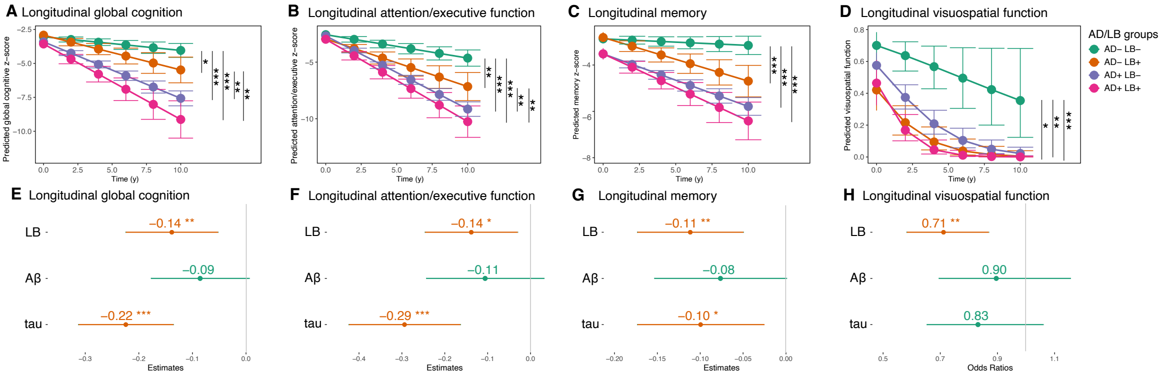

Supplementary Figure 2A-H. Comparisons between AD/LB groups and the independent effects of LB, Aβ and tau pathologies on longitudinal cognitive function with corrections for multiple comparisons. In **A-D**, group comparisons were performed using mixed-effects models examining the AD/LB group * time interaction, adjusted for age, sex, education, and cognitive stage (MCI/dementia). In **E-H**, the interaction time * all three pathologies (binarized) were examined in the same model adjusted for age, sex, and education, to examine the independent effect of pathology and cognitive progression. Outcomes were z-scored cognitive tests according to the distribution in Aβ-negative controls. **A-D** show estimated marginal means and 95% confidence interval of the means obtained from linear mixed-effects models by AD/LB group. In **E-H**, the dot/center shows the interaction estimates of time * pathology and error bars represent the 95% confidence interval. In **C** and **G**, binomial mixed-effects models were used since the outcome was binary. 302 participants were AD-/LB-, 106 AD-/LB+, 377 AD+/LB-, and 98 AD+/LB+. 204 were LB+, 607 Aβ+, and 489 tau+. Multiple comparison corrections were applied per outcome using the FDR method (at α=0.05). Missing data are shown in Supplementary Table 2.
* $p<0.05$; ** $p<0.01$; *** $p<0.001$

SUPPLEMENTARY TABLES

Supplementary Table 1. Missing data in cross-sectional analyses (n=883)

**Variables**	**Missingness**
Global cognitive function[1]	37.6%
Memory function[2]	6.5%
Attention/executive function[3]	1.3%
Visuospatial function[4]	1.4%
Symbol Digit Modalities Test	28.2%
MMSE Serial 7s	1.9%
Presence of hallucinations (CIMP-QUEST)	30.2%
Motor function (CIMP-QUEST)	24.2%

[1] Measured using the mPACC5
[2] Measured using the 10-word delayed recall test from ADAS-cog
[3] Measured using the Symbol Digit Modalities Test if present, otherwise using the serial 7s subscore from the MMSE.
[4] Measured using the VOSP incomplete letters task in BioFINDER-2 and pentagon copying from the MMSE in BioFINDER-1.

Supplementary Table 2. Missing data in longitudinal analyses

Variables	Overall (n=3,173 data points)	Baseline (n=883)	1-year (n=730)	2-year (n=677)	3-year (n=349)	4-year (n=263)	5-year (n=104)	6-year (n=126)
Global cognitive function[1]	56.2%	37.6%	70.4%	55.4%	69.6%	54.8%	82.7%	59.2%
Memory function[2]	19.4%	6.5%	23.0%	26.1%	22.9%	23.2%	30.8%	24.6%
Attention/executive function[3]	5.0%	1.3%	4.0%	5.9%	7.2%	9.5%	15.4%	10.3%
Visuospatial function[4]	5.3%	1.4%	3.8%	5.9%	6.9%	10.7%	17.3%	14.3%
Symbol Digit Modalities Test	43.6%	28.2%	50.6%	51.1%	46.1%	45.6%	62.5%	48.4%
MMSE Serial 7s	5.8%	1.9%	4.3%	6.9%	8.0%	10.3%	17.3%	12.7%

[1] Measured using the mPACC5
[2] Measured using the 10-word delayed recall test from ADAS-cog
[3] Measured using the Symbol Digit Modalities Test if present, otherwise using the serial 7s subscore from the MMSE.
[4] Measured using the VOSP incomplete letters task in BioFINDER-2 and pentagon copying from the MMSE in BioFINDER-1.

Supplementary Table 3. Diagnostic classification of Parkinson's disease

PD case	Level of certainty of baseline diagnosis (Gelb et al.[47])	Level of certainty of baseline diagnosis (MDS criteria[15])	Level of certainty of longitudinal diagnosis (Gelb et al.[47])	Level of certainty of longitudinal diagnosis (MDS criteria[15])
1	Possible	Probable	Possible	Established
2	Probable	Established	Probable	Established
3	Probable	Established	Probable	Established
4	Possible	Probable	Possible	Established
5	Probable	Established	Probable	Established
6	Probable	Established	Probable	Established
7	Possible	Established	Possible	Established
8	Not fulfilling criteria	Not fulfilling criteria	Possible	Probable
9	Probable	Established	Probable	Established
10	Possible	Probable	Possible	Probable
11	Possible	Probable	Probable	Probable
12	Probable	Established	Probable	Established
13	Possible	Probable	Possible	Probable
14	Probable	Established	Probable	Established
15	Not fulfilling criteria	Not fulfilling criteria	Possible	Established
16	Possible	Probable	Possible	Probable
17	Possible	Probable	Probable	Established

Longitudinal diagnosis refers to fulfilling the diagnostic criteria either at baseline or during subsequent follow-up visits. Cases with either possible or probable PD according to Gelb et al. were classified as PD in the current study.
