# Supplementary material for: Clinical effects of Lewy body pathology in cognitively impaired individuals
Source: Nat Med. 2023 Jul 18;29(8):1964–70. doi: 10.1038/s41591-023-02449-7 (PMC10427416; doi:10.1038/s41591-023-02449-7)
Supplement: Supplementary file 2 — Reporting Summary [file 41591_2023_2449_MOESM2_ESM.pdf]

Corresponding author(s): Piero Parchi
Oskar Hansson

Last updated by author(s): May 30, 2023

# Reporting Summary

Nature Portfolio wishes to improve the reproducibility of the work that we publish. This form provides structure for consistency and transparency in reporting. For further information on Nature Portfolio policies, see our Editorial Policies and the Editorial Policy Checklist.

## Statistics

For all statistical analyses, confirm that the following items are present in the figure legend, table legend, main text, or Methods section.

| n/a | Confirmed | |
|---|---|---|
| ☐ | ☒ | The exact sample size (*n*) for each experimental group/condition, given as a discrete number and unit of measurement |
| ☐ | ☒ | A statement on whether measurements were taken from distinct samples or whether the same sample was measured repeatedly |
| ☐ | ☒ | The statistical test(s) used AND whether they are one- or two-sided<br>*Only common tests should be described solely by name; describe more complex techniques in the Methods section.* |
| ☐ | ☒ | A description of all covariates tested |
| ☐ | ☒ | A description of any assumptions or corrections, such as tests of normality and adjustment for multiple comparisons |
| ☐ | ☒ | A full description of the statistical parameters including central tendency (e.g. means) or other basic estimates (e.g. regression coefficient) AND variation (e.g. standard deviation) or associated estimates of uncertainty (e.g. confidence intervals) |
| ☐ | ☒ | For null hypothesis testing, the test statistic (e.g. *F*, *t*, *r*) with confidence intervals, effect sizes, degrees of freedom and *P* value noted<br>*Give P values as exact values whenever suitable.* |
| ☒ | ☐ | For Bayesian analysis, information on the choice of priors and Markov chain Monte Carlo settings |
| ☒ | ☐ | For hierarchical and complex designs, identification of the appropriate level for tests and full reporting of outcomes |
| ☒ | ☐ | Estimates of effect sizes (e.g. Cohen's *d*, Pearson's *r*), indicating how they were calculated |

*Our web collection on statistics for biologists contains articles on many of the points above.*

## Software and code

Policy information about availability of computer code

| | |
|---|---|
| Data collection | No software was used. |
| Data analysis | R version 4.1 using packages lme4 (ver 1.1) and lmerTest (ver 3.1). |

For manuscripts utilizing custom algorithms or software that are central to the research but not yet described in published literature, software must be made available to editors and reviewers. We strongly encourage code deposition in a community repository (e.g. GitHub). See the Nature Portfolio guidelines for submitting code & software for further information.

## Data

Policy information about availability of data

All manuscripts must include a data availability statement. This statement should provide the following information, where applicable:

- Accession codes, unique identifiers, or web links for publicly available datasets
- A description of any restrictions on data availability
- For clinical datasets or third party data, please ensure that the statement adheres to our policy

Anonymized data will be shared by request from a qualified academic investigator for the sole purpose of replicating procedures and results presented in the article and as long as data transfer is in agreement with EU legislation on the general data protection regulation and decisions by the Ethical Review Board of Sweden and Region Skåne, which should be regulated in a material transfer agreement.

## Human research participants

Policy information about studies involving human research participants and Sex and Gender in Research.

| | |
|---|---|
| Reporting on sex and gender | The study includes both men and women. Sex was assigned (not self reported). 404 (45.8%) were female. Sex was adjusted for in the statistical models. |
| Population characteristics | Detailed information is given in Table 1, Extended Data Table 1 and Online Methods. In short, we present results for analyses from the BioFINDER-1 and BioFINDER-2 cohorts, both with very similar demographics. Cognitively impaired participants were included in the present study (n=481 (54.5%) with MCI and 402 (45.5%) with dementia) with cross-sectional and longitudinal data. The mean (SD) age was 73 (7.2) years. |
| Recruitment | This project was done as part of the prospective Swedish BioFINDER study. All patients were recruited from the Southern part of Sweden and underwent baseline examination from 2007 to 2015 (BioFINDER-1) or from 2017 to 2021 (BioFINDER-2). In BioFINDER-1, participants were consecutively recruited based on referrals (mostly from primary care) to participating memory clinics (in the towns of Malmö, Lund and Ängelholm in Sweden). In BioFINDER-2, patients were included after being referred to the memory clinic of Skåne University Hospital in Malmö, Sweden. Details of the recruitment is described in reference 34 and Methods-only references 2-3. Per study design, only those that speak Swedish were included, which might reduce the number of foreign-born participants. |
| Ethics oversight | The study was approved by the Regional Ethics Committee in Lund, Sweden. All participants gave their informed consent to participate in the study and the data were collected according to the Declaration of Helsinki. |

Note that full information on the approval of the study protocol must also be provided in the manuscript.

# Field-specific reporting

Please select the one below that is the best fit for your research. If you are not sure, read the appropriate sections before making your selection.

☒ Life sciences ☐ Behavioural & social sciences ☐ Ecological, evolutionary & environmental sciences

For a reference copy of the document with all sections, see nature.com/documents/nr-reporting-summary-flat.pdf

# Life sciences study design

All studies must disclose on these points even when the disclosure is negative.

| | |
|---|---|
| Sample size | The study included a large sample size (n=883 participants with cross-sectional data and 3,173 data points for the longitudinal analysis). This also included large samples in the different subgroups: AD-/LB- (N=302), AD-/LB+ (N=106), AD+/LB- (N=377), and AD+/LB+ (N=98) as well as for the prevalence of LB (n=204, 23.1%), amyloid (n=607, 69%), and tau (n=489, 55%). Power analyses indicated that the study was powered to detect small effect sizes (at power=80%, $\alpha$=0.05). |
| Data exclusions | Analyses were performed on all eligible participants and not on a restricted sample with complete data for all cognitive and non-cognitive measures. The rationale for this was to not introduce a selection bias. |
| Replication | Two independent cohorts were used in the study. Data was pooled to achieve better statistical power and no replication in a third cohort was perform. |
| Randomization | In these 2 cohort studies (observational studies) no allocation into experimental groups were performed, therefore randomization is not relevant to this study. Statistical analyses were controlled for potential confounding effects of age, sex and education. |
| Blinding | Diagnostic assessments and all test measures were performed blinded to the α-synuclein SAA results. Group allocations (AD/LB groups) were performed after all clinical assessments (hence blinded). |

# Reporting for specific materials, systems and methods

We require information from authors about some types of materials, experimental systems and methods used in many studies. Here, indicate whether each material, system or method listed is relevant to your study. If you are not sure if a list item applies to your research, read the appropriate section before selecting a response.

## Materials & experimental systems

| n/a | Involved in the study | |
|---|---|---|
| ☐ | ☒ | Antibodies |
| ☒ | ☐ | Eukaryotic cell lines |
| ☒ | ☐ | Palaeontology and archaeology |
| ☒ | ☐ | Animals and other organisms |
| ☐ | ☒ | Clinical data |
| ☒ | ☐ | Dual use research of concern |

## Methods

| n/a | Involved in the study | |
|---|---|---|
| ☒ | ☐ | ChIP-seq |
| ☒ | ☐ | Flow cytometry |
| ☒ | ☐ | MRI-based neuroimaging |

# Antibodies

Antibodies used

Phosphorylated tau 217( p-tau217) assay.

Validation

The phosphorylated tau 217 ( p-tau217) assay in CSF was performed using phospho-specific biotinylated capture antibody (IBA493, developed by Lilly Research Laboratories) and SULFO-TAG- conjugated anti-tau detection antibody (4G10E2, developed by Lilly Research Laboratories). The p-tau217 immunoassay has been fully described by Palmqvist et al. (JAMA. 2020;324(8):772-781).

# Clinical data

Policy information about clinical studies

All manuscripts should comply with the ICMJE guidelines for publication of clinical research and a completed CONSORT checklist must be included with all submissions.

Clinical trial registration

NCT01208675 and NCT03174938

Study protocol

BioFINDER-1: https://clinicaltrials.gov/ct2/show/NCT01208675
BioFINDER-2: https://clinicaltrials.gov/ct2/show/NCT03174938

Data collection

All patients were recruited from the Southern part of Sweden and underwent baseline examination from 2007 to 2015 (BioFINDER-1) or from 2017 to 2021 (BioFINDER-2) at a memory clinic setting (Skåne University Hospital and the hospital of Ängelholm). Biomarker data was collected at baseline.

Outcomes

Primary outcomes were cognitive test scores (global cognition, attention/executive function, memory, and visuospatial function), and motor function (using the CIMP-QUEST questionnaire).
